# Challenges in the culture-independent analysis of oral and respiratory samples from intubated patients

**DOI:** 10.3389/fcimb.2014.00065

**Published:** 2014-05-23

**Authors:** Vladimir Lazarevic, Nadia Gaïa, Stéphane Emonet, Myriam Girard, Gesuele Renzi, Lena Despres, Hannah Wozniak, Javier Yugueros Marcos, Jean-Baptiste Veyrieras, Sonia Chatellier, Alex van Belkum, Jérôme Pugin, Jacques Schrenzel

**Affiliations:** ^1^Genomic Research Laboratory, Department of Genetics and Laboratory Medicine and Department of Medical Specialties, Geneva University HospitalsGeneva, Switzerland; ^2^Clinical Microbiology Laboratory, Department of Genetics and Laboratory Medicine and Department of Medical Specialties, Geneva University HospitalsGeneva, Switzerland; ^3^Department of Internal Medicine, Rehabilitation and Geriatrics, Geneva University HospitalsGeneva, Switzerland; ^4^Medical Diagnostic Discovery Department, BioMérieuxGrenoble, France; ^5^Data and Knowledge Laboratory, BioMérieuxMarcy l'Etoile, France; ^6^Research and Development Microbiology, BioMérieuxLa Balme-les-Grottes, France; ^7^Laboratory of Intensive Care, Department of Anaesthesiology, Pharmacology and Intensive Care, Geneva University HospitalsGeneva, Switzerland

**Keywords:** endotracheal aspirate, supraglottic secretions, microbiota, 16S rDNA profiling, bacterial communities

## Abstract

The spread of microorganisms in hospitals is an important public health threat, and yet few studies have assessed how human microbial communities (microbiota) evolve in the hospital setting. Studies conducted so far have mainly focused on a limited number of bacterial species, mostly pathogenic ones and primarily during outbreaks. We explored the bacterial community diversity of the microbiota from oral and respiratory samples of intubated patients hospitalized in the intensive care unit and we discuss the technical challenges that may arise while using culture-independent approaches to study these types of samples.

## Subjects and sample types

Supraglottic secretions (SGS) and endotracheal aspirates (ETA) were collected on a daily basis from five subjects over a 4-day period following intubation (Supplementary Material). For each subject, we also included a sample on day 5–11 depending on availability. None of the patients developed ventilator-associated pneumonia (VAP), so their oral/respiratory bacterial communities likely represent “healthy” microbiota of mechanically ventilated patients.

## Sequence data processing

After pyrosequencing of 16S rDNA V1-3 amplicon libraries from the reverse primer (Supplementary Material), a total of 383,302 sequence reads had an exact match to the barcode sequence. Removal of sequence reads based on (1) the match to the 16S rDNA sequence of the reverse primer, (2) length, (3) quality score, (4) the presence of homopolymer runs and (5) ambiguous bases, resulted in 375,612 (98%), 375,210 (99.8%), 266,338 (69.5%), 266,338 (69.5%), and 264,358 (69%) sequences, respectively. The BLASTN-based OTU picking, performed as described previously (Lazarevic et al., 2013a) using the Greengenes taxonomy (McDonald et al., 2012), further reduced the dataset to 217,531 sequences (56.8%) of which 209,477 derived from the 50 clinical (25 SGS and 25 ETA) samples and 8054 sequence reads corresponded to 8 negative controls (reagents). After removal of possibly contaminant 16S rDNA sequences (see below) the sample dataset was represented by 194,322 sequences. The number of sequences per sample varied between 9 and 7665 (average 3886, median 4500). The average number of sequences per individual were 24,712 (median 22,763) and 14,153 (median 15,082) for SGS and ETA, respectively.

## Microbiota profiles

The phyla Firmicutes, Proteobacteria, Bacteroidetes, Fusobacteria, Tenericutes and Actinobacteria were highly prevalent (42–50 positive samples) and corresponded on average to >97% of the 16S rDNA sequences in both SGS and ETA samples datasets. The less abundant phyla Spirochaetes, TM7 and Synergistetes were also identified in both samples types (24–30 positive samples). Other phyla, SR1, Cyanobacteria, Thermi and WPS-2 were found each in less than 5 samples at low proportion (<0.2%). A total of 115 genera were identified in the dataset. At the genus level, SGS and ETA microbiota showed high similarity in terms of the prevalence (Pearson *R* = 0.884) and average relative abundance (Pearson *R* = 0.854). Genera *Streptococcus, Neisseria*, and *Prevotella* had the highest proportion in both samples types and represented together 56 and 57% of sequence reads in SGS and ETA, respectively. In contrast, *Mycobacterium*, the fourth most abundant genus in SGS (6.7%) and ETA (3.1%) has been identified in saliva at very low levels (Lazarevic et al., 2011). Interestingly, in a study which included intubated patients, the genus *Mycoplasma* was found in bronchoalveolar lavages but only in individuals who developed VAP or community associated pneumonia (Bousbia et al., 2012). The other genus from the phylum Tenericutes, *Ureaplasma*, was frequently identified as dominant organism in tracheal aspirates from mechanically ventilated preterm infants (Mourani et al., 2011).

**Figure 1:**
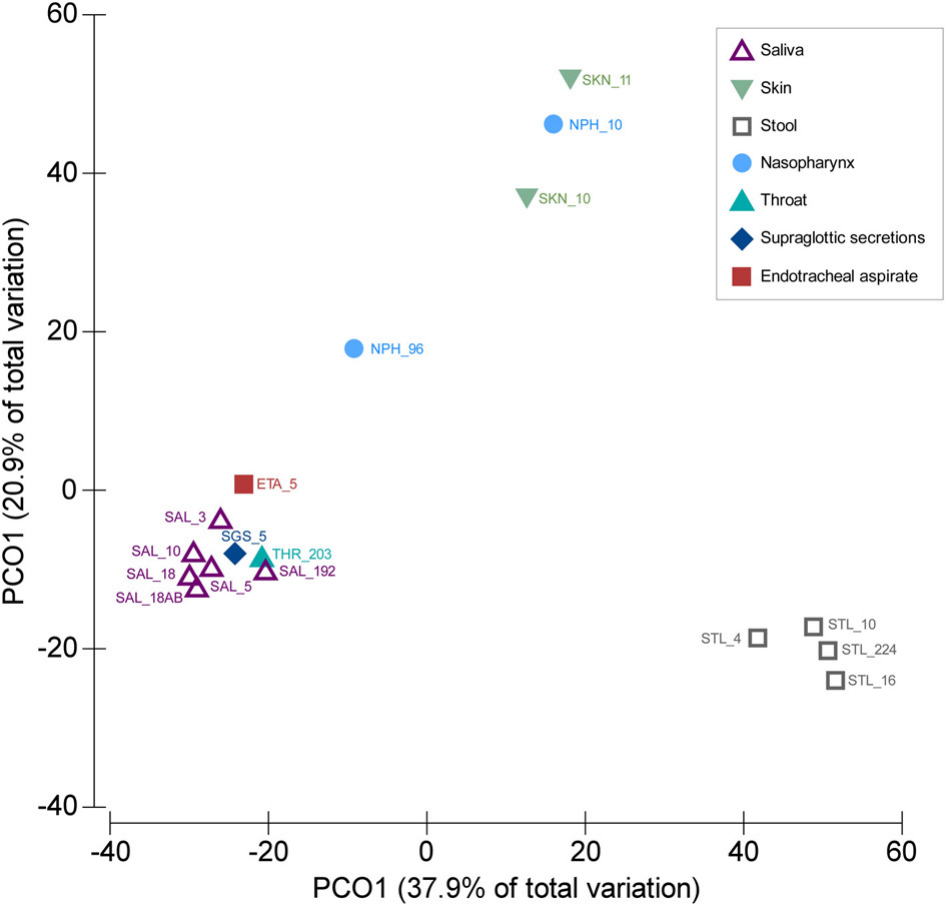
**Similarities between SGS, ETA, and bacterial communities from other body parts**. PCoA was based on Bray–Curtis similarity matrix constructed using square-root transformed average relative abundance of genera. The number following underscore corresponds to the number of subjects analyzed. Saliva samples: SAL_192 (Segata et al., 2012), SAL_10 (Ling et al., 2013), SAL_18 (from children, before antibiotic treatment) (Lazarevic et al., 2013b), SAL_18AB (from children, at the end of the antibiotic treatment) (Lazarevic et al., 2013b), SAL_5 (three time points for each subject) (Lazarevic et al., 2010), SAL_3 (Zaura et al., 2009). Throat swab: THR_203 (Segata et al., 2012); Supraglottic secretions: SGS_5 (five time points for each subject, this study); Endotracheal aspirate: ETA_5 (five time points for each subject, this study). Skin samples: SKN_10 (Ling et al., 2013), SKN_11 (MG-RAST ID 6526); Nasopharyngeal swabs: NPH_96 (Bogaert et al., 2011), NPH_10 (Ling et al., 2013). Stool samples: STL_224 (Segata et al., 2012), STL_10 (Ling et al., 2013), STL_16 (five time points for each subject) (Krych et al., 2013), STL_4 (data designated V4-0.5) (Claesson et al., 2009). The abundance of genera were taken directly from the tables provided in corresponding publications, except for the SKN_11 (unpublished) for which the MG-RAST data were processed using the bioinformatics pipeline as described in this paper.

We compared the SGS and ETA microbiota with those from other body sites available from published studies. Both SGS and ETA bacterial communities determined in our study clustered together with salivary (Zaura et al., 2009; Lazarevic et al., 2010, 2013b; Segata et al., 2012; Ling et al., 2013) and throat (back wall of oropharynx) microbiota (Segata et al., 2012), and were clearly distinct from the skin microbiota (Ling et al., 2013) (unpublished, MG-RAST ID 6526), nasopharyngeal microbiota (Bogaert et al., 2011; Ling et al., 2013) and gut microbiota (Claesson et al., 2009; Segata et al., 2012; Krych et al., 2013; Ling et al., 2013) (Figure 1). This significantly supports the validity of our experimental approach and shows that differences between anatomical sites outweighed the methodological differences related to DNA extraction, PCR amplification and bioinformatics analysis. Our result is consistent with the recent metagenomic studies indicating that the lower respiratory tract microbiota (including trachea) originate mainly from the oral and upper respiratory tract (including oropharynx) in healthy subjects and in disease (Charlson et al., 2011; Cabrera-Rubio et al., 2012; Segata et al., 2012). Similarly, culture-based studies showed that bacterial communities of the pharyngeal and tracheal secretions are similar (Pirracchio et al., 2009).

## Technical challenges

Since many bacteria are not readily cultivable, the studies of bacterial communities using culture-independent methods provide a benefit over the traditional approaches in which bacterial identification requires growth under laboratory conditions. However, culture-free molecular methods introduce biases related to: DNA extraction procedure, PCR amplification, sequencing platform used, and bio-informatic analysis (Lazarevic et al., 2013a; Lozupone et al., 2013). Below we discuss some of the challenges in the culture-independent analysis of SGS and ETA related to the physical and microbiological nature of these samples.

### Viscosity of samples

Because of the high viscosity observed in about 5% of ETA samples, we added dithiothreitol (DTT) in the lysis buffer for DNA extraction. By dissolving mucus, DTT treatment liquefies samples (Olsson et al., 1993) and allows for further and optimized sample processing in a semi-automated workflow. DTT and other thiol-reducing agents used to reduce *in vitro* viscosity of the mucin (Sheffner, 1963) have the potential to inhibit the subsequent PCR amplification (Deneer and Knight, 1994). We performed DNA extraction with or without DTT addition for six ETA samples and we found that 16S rDNA amplicon yields were higher when the extraction procedure included DTT-treatment (not presented). Therefore, DTT may be systematically added to viscous samples provided that it is efficiently washed away before the PCR step, as evidenced in these conditions.

### Co-extraction of bacterial and human DNA

**Figure 2:**
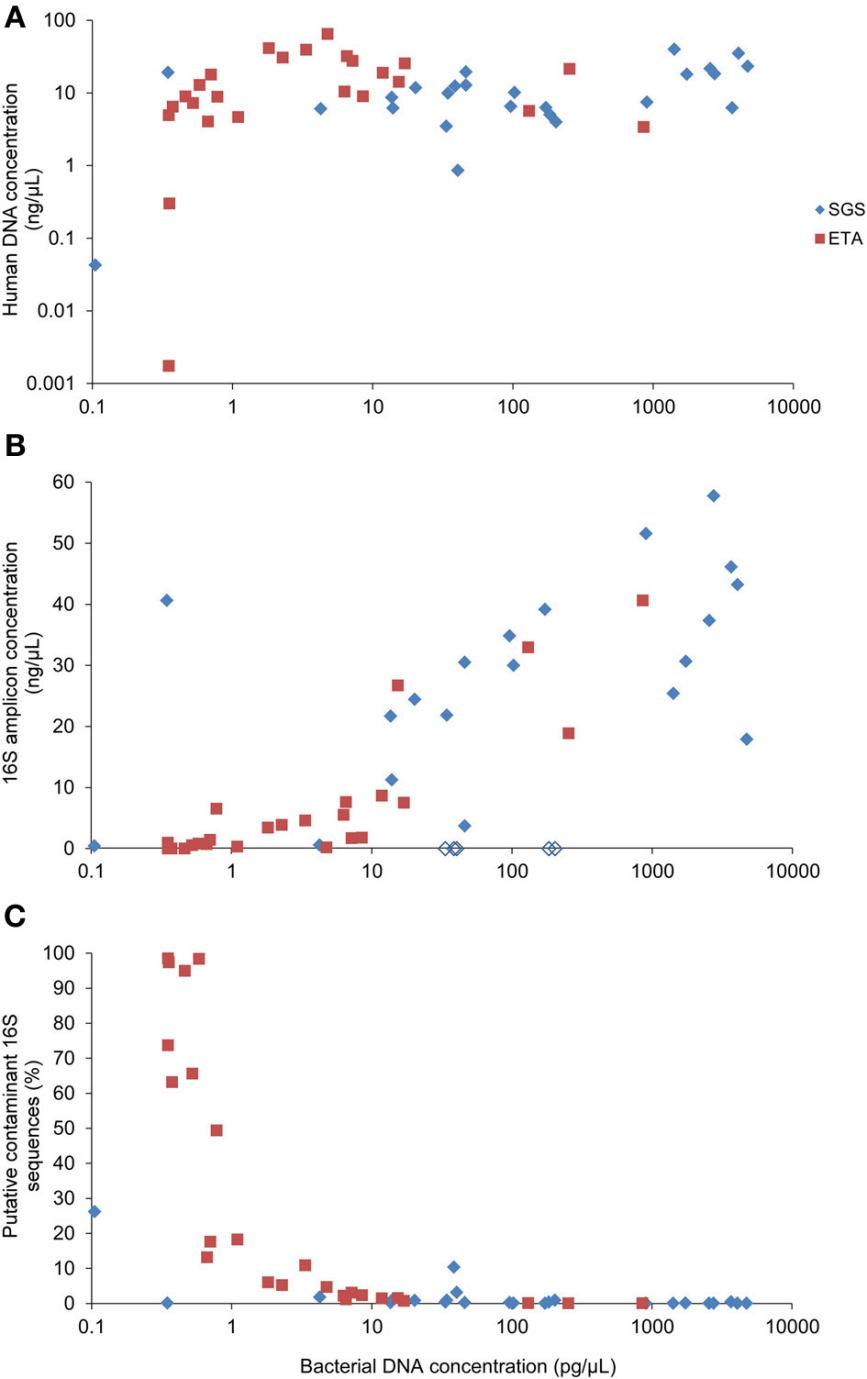
**Correlation between bacterial DNA yield and (A) human DNA yield, (B) concentration of the 16S rDNA amplicons or (C) percentage of putative contaminant 16S rDNA sequences**. Bacterial and human DNA concentration in purified extracts was determined by qPCR. The yield of the 16S rDNA amplicon concentration was determined using Bioanalyzer. Empty lozenges (in **B**) correspond to SGS samples from the subject #5.

Real-time PCR revealed that the bacterial DNA concentration in extracts varied from 0.1 to 4723 pg/μL with median values of 96.2 and 2.3 pg/μL for SGS and ETA, respectively. The yield of human DNA was generally much higher (median 10.1 ng/uL) and showed less variation in concentration (Figure 2A). Grice et al. (2008) showed that a mixture of human and bacterial DNA in up to a 100,000:1 mass ratio (100:1 cell ratio) did not significantly alter 16S rDNA amplification. However, the presence of human DNA is more critical when a whole genome shotgun sequencing approach is to be used to study microbial communities because many reads will derive from host DNA. To circumvent this potential limitation, a method for selective enrichment of microbial DNA from contaminating human host DNA has been developed (Feehery et al., 2013), but further progress is needed in this area. Alternatively, host DNA sequences may be recognized and removed by bio-informatic analysis (Schmieder and Edwards, 2011).

### PCR inhibition

We observed that all of the five SGS samples from one patient (#5) presumably contained PCR inhibitors. Figure 2B shows that non-diluted SGS samples from this subject did not produce measurable amounts of PCR products. A 100-fold sample dilution was required to obtain a visible band of the 16S rDNA V1-3 amplicon upon electrophoresis. However, sample dilution may not be optimal in cases where the bacterial concentration is low, as it may reduce already low input DNA. The qPCR analysis of the samples from patient #5 performed after serial dilution did not reveal the inhibitory effect (not presented). This is in line with evidence that PCR inhibition depends on amplification conditions and the DNA polymerase being used (Al-Soud and Rådström, 1998). Therefore, to reduce PCR inhibition, it may be helpful to use genetically engineered DNA polymerases highly tolerant to inhibition (Kermekchiev et al., 2009).

### Contaminant DNA

Reagents used for DNA extraction and PCR may contain bacterial DNA which is overruled by DNA from high-density samples. However, sequence reads derived from samples with low DNA concentration may largely originate from exogenous DNA contamination.

We included in the pyrosequencing run the PCR amplification products obtained using eight negative controls. Any operational taxonomic unit (OTU) that had greater average relative abundance in negative controls than in clinical samples was considered as contaminant. The proportion of putative contaminant 16S rDNA sequences was inversely correlated with bacterial DNA concentration in DNA extracts (Spearman *r* = −0.850) (Figure 2C). Most contaminating OTUs (93/127) were assigned to Proteobacteria, already identified before as common reagent contaminants (Tanner et al., 1998; Biesbroek et al., 2012; Willner et al., 2012). The sequence reads assigned to the putative contaminating OTUs represented 7.2% of the reads in the sample dataset. Processing of the sequence datasets using a minimum identity threshold of 99% and the reference OTU database pre-clustered at 99% resulted in only slightly higher proportion (8%) of putative contaminating sequences. However, distinction between putative contaminants and “true” sequences will remain an important variable in metagenomic approaches.

### Low DNA yield

It remains unclear whether prophylactic chlorhexidine oral rinse, given to all patients in our study, decreases total bacterial load in the trachea as it has been the case with saliva (Veksler et al., 1991). 16S rDNA amplicon libraries deriving from the samples with very low bacterial load resulted in a low number of sequence reads. DNA extraction using larger sample volume (if available) and/or concentration of bacteria by centrifugation may provide a solution. Performing additional PCR cycles in order to increase the amplicon yield has been shown to introduce amplification biases in salivary samples (Lazarevic et al., 2012). Another strategy to cope with low DNA concentration is the use of multiple displacement amplification (MDA) prior to 16S rDNA amplification (Pragman et al., 2012), but MDA may also introduce a representational bias (Marine et al., 2014).

## Outlook

In this pilot study, involving a small number of intubated patients, we pointed to some common issues that may arise when analysing their oropharyngeal and respiratory-tract microbiota. We provided a preliminary characterization of the microbiota associated with these specific sample types that have been only weakly (ETA) or not at all (SGS) studied so far using culture-independent methods. The analysis of larger cohorts of intubated patients with a longer follow-up period may allow to (1) answer whether the oropharyngeal and respiratory microbiota from different patients converge to one or several distinct states during hospitalization and to (2) link microbiome structure to the development of VAP which occurs in up to 30% of patients receiving mechanical ventilation (Morrow et al., 2010).

### Conflict of interest statement

This investigation-driven study was financially supported by BioMérieux. The four authors are employees of BioMérieux, a company creating and developing infectious disease diagnostics. The authors declare that the research was conducted in the absence of any commercial or financial relationships that could be construed as a potential conflict of interest.
