# Supplementary material for: Challenges in the culture-independent analysis of oral and respiratory samples from intubated patients
Source: Front Cell Infect Microbiol. 2014 May 23;4:65. doi: 10.3389/fcimb.2014.00065 (PMC4033159; doi:10.3389/fcimb.2014.00065)
Supplement: Supplementary file 1 [file DataSheet1.DOCX]

**SUPPLEMENTARY TEXT**

**Methods**

***Subject recruitment and sampling***

This study was approved by the Ethics Committee of the Geneva University Hospitals (CER 12-051/NAC 12-023). Written informed consent of relatives was obtained, and whenever possible (after extubation) patient’s informed consent was also obtained.

We included intubated patients from the Intensive Care Unit (ICU) of the Geneva University Hospital. Inclusion criteria were: patients hospitalised in the adult ICU (>18 years old), who had been intubated in the last 24 hours, with an expected duration of intubation >4 days (e.g. neurologic disease or trauma). Excluded were patients for whom regular tracheal aspirations could not be performed (because of unstable intracranial pressure, for example), or whom received an antibiotic therapy in the week preceding the intubation. Chlorhexidine mouthwash was given daily to all patients.

SGS and ETA were collected daily, starting in the first 24 hours after intubation from day 1 (first sample) to day 4. In addition, a sample from day 5 (patients #2 and #5), 10 (patient #1) and 11 (patients #3 and #4) were included. The samples were collected directly, or after instillation of sterile saline (0.9% NaCl) when needed for ETA. Samples were kept refrigerated (4°C) after collection and frozen at -80°C within 24 h.

***DNA extraction***

An aliquot of around 400-µL of each sample was mixed with 700 µL of the lysis buffer (20 mM Tris, 2 mM EDTA pH8, 1% Tween-20, 2% TritonX-100) and 50 µL 1M DTT. The mixture was shaken in a NucleoSpin Bead Tube containing ceramic beads (Macherey-Nagel) for 4 min at maximum speed on a Vortex-Genie 2 with a horizontal tube holder (Scientific Industries). DNA was extracted and purified from 800 µL of the supernatant using the easyMag System (bioMérieux), following a specific protocol with 50 uL silica and an elution volume of 40 µL. Purified DNA was stored at -20°C. Eight negative control samples were performed by using 400 µL of water (in triplicate), lysis buffer (in triplicate) or saline (in duplicate), following the same extraction procedure as described above for clinical specimens.

We amplified the V1-3 region of the bacterial 16S rDNA, corresponding to *Escherichia coli* 16S rDNA positions 28-514 (excluding primer sequences). The PCRs included 11.5 µL of DNA extract and 12 pmol of each forward (5’-ctatgcgccttgccagcccgctcag*ac*GAGTTTGATCMTGGCTCAG) and a barcoded reverse primer (5’-cgtatcgcctccctcgcgccatcagNNNNNNNN*at*CCGCGGCTGCTGGCAC) ([Lazarevic et al., 2013](#_ENREF_3)) in 25 µL Primestar HS Premix (Takara). For each sample, duplicate PCRs were carried out for 30 cycles using the following parameters: 98°C for 10 s, 56°C for 15 s, 72°C for 1 min. Two replicate PCRs were then pooled. DNA concentration of amplicons of interest was determined on the 2100 Bioanalyzer (Agilent Technologies) using a DNA1000 lab chip. Each sample was column-purified by using the QIAquick PCR purification kit (Qiagen, Hilden, Germany) and eluted in 30 µL water. Thirty ng amplicon DNA from each sample was pooled. For the samples with <30 ng DNA in the eluate, the entire eluate was added to the pool. Twenty µL eluate was used for the negative control samples. The 454 GS FLX Titanium pyrosequencing from the Adaptor A (reverse primer) side was performed at LGC (Berlin, Germany).

***qPCR***

To determine the concentration of bacterial and human DNA we performed qPCR experiments as described previously ([Lazarevic et al., 2013](#_ENREF_3)), using 16S rRNA and beta-actin reference genes, respectively. The reference curves for bacterial and human DNA quantitation were created using known concentrations of *Staphylococcus aureus* MW2 genomic DNA and human genomic DNA from the TaqMan beta-Actin Detection Reagent kit (Applied Biosystems), respectively.

***Sequence analysis***

Sequence filtering was performed using the command 'trim.seqs' from the mothur (v 1.32) software package ([Schloss et al., 2009](#_ENREF_5)). We removed sequences that met any of the following criteria: (i) contained a mismatch in the barcode or contained more than one mismatch in the 16S rDNA sequence of the reverse primer; (ii) contained ambiguous bases; (iii) were <250 or >600 nt in length after trimming of the reverse primer sequence; (iv) contained runs of ≥10 identical nucleotides; (v) had a minimum quality score <35 over a 50-nt window. Any complete or residual sequences of the forward PCR primer were removed after sequence alignment (mothur command 'align.seqs') against the Greengenes reference database ([McDonald et al., 2012](#_ENREF_4)). The sequences that passed the step (i) were combined with quality data and deposited in MG-RAST as a fastq file under the accession number 4552116.3. Taxonomic assignments of phylotypes were made by OTU picking with the Greengenes reference database ([McDonald et al., 2012](#_ENREF_4)) pre-clustered at 97% using BLASTN (-evalue 1e-50 -perc_identity 97 -max_target_seqs 1) ([Altschul et al., 1990](#_ENREF_1)) and a minimum query alignment coverage of 90%, as described previously in 'Pipeline 6' ([Lazarevic et al., 2013](#_ENREF_3)). Downstream analyses were made using taxonomic information of the matching full length 16S rDNA sequences. OTUs identified in negative controls with an average relative abundance higher to that found in SGS/ETA samples were subtracted from the sample dataset prior to further analysis. For comparison purposes, we also performed taxonomic assignments by OTU picking with the Greengenes reference database pre-clustered at 99% and minimum identity of 99%.

To compare bacterial communities, we constructed a Bray-Curtis ([Bray and Curtis, 1957](#_ENREF_2)) similarity matrix based on the square-root transformed relative abundance of genera. Principal coordinates analysis (PCoA) of Bray-Curtis similarities was performed in PRIMER-E (Primer-E Ltd., Plymouth, UK).

**References**

Altschul, S.F., Gish, W., Miller, W., Myers, E.W., and Lipman, D.J. (1990). Basic local alignment search tool. *J Mol Biol* 215**,** 403-410.

Bray, R., and Curtis, J.T. (1957). An ordination of the upland forest communities of southern Wisconsin. *Ecological Monographs* 27**,** 325-334.

Lazarevic, V., Gaia, N., Girard, M., Francois, P., and Schrenzel, J. (2013). Comparison of DNA extraction methods in analysis of salivary bacterial communities. *PLoS ONE* 8**,** e67699.

Mcdonald, D., Price, M.N., Goodrich, J., Nawrocki, E.P., Desantis, T.Z., Probst, A., Andersen, G.L., Knight, R., and Hugenholtz, P. (2012). An improved Greengenes taxonomy with explicit ranks for ecological and evolutionary analyses of bacteria and archaea. *The ISME Journal* 6**,** 610-618.

Schloss, P.D., Westcott, S.L., Ryabin, T., Hall, J.R., Hartmann, M., Hollister, E.B., Lesniewski, R.A., Oakley, B.B., Parks, D.H., Robinson, C.J., Sahl, J.W., Stres, B., Thallinger, G.G., Van Horn, D.J., and Weber, C.F. (2009). Introducing mothur: open-source, platform-independent, community-supported software for describing and comparing microbial communities. *Appl Environ Microbiol* 75**,** 7537-7541.
